# Prediction Model of Extubation Outcomes in Critically Ill Patients: A Multicenter Prospective Cohort Study

**DOI:** 10.3390/jcm11092520

**Published:** 2022-04-29

**Authors:** Aiko Tanaka, Daijiro Kabata, Osamu Hirao, Junko Kosaka, Nana Furushima, Yuichi Maki, Akinori Uchiyama, Moritoki Egi, Ayumi Shintani, Hiroshi Morimatsu, Satoshi Mizobuchi, Yoshifumi Kotake, Yuji Fujino

**Affiliations:** 1 Department of Anesthesiology and Intensive Care Medicine, Osaka University Graduate School of Medicine, 2-15 Yamadaoka, Suita 565-0871, Japan; auchiyama@hp-icu.med.osaka-u.ac.jp (A.U.); yujif217@gmail.com (Y.F.); 2 Department of Medical Statistics, Graduate School of Medicine, Osaka Metropolitan University, 1-4-3 Asahimachi, Abeno-ku, Osaka 545-8585, Japan; daijiro.kabata@omu.ac.jp (D.K.); ayumi.shintani@omu.ac.jp (A.S.); 3 Department of Anesthesiology, Osaka General Medical Center, 3-1-56 Bandai-Higashi, Sumiyoshi-ku, Osaka 558-8558, Japan; hirahira1985@yahoo.co.jp; 4 Department of Anesthesiology and Resuscitology, Okayama University Hospital, 2-5-1 Shikata-cho, Kita-ku, Okayama 700-8558, Japan; junkok@okayama-u.ac.jp (J.K.); pb9b45wr@okayama-u.ac.jp (H.M.); 5 Department of Anesthesiology and Intensive Care Medicine, Kobe University Hospital, 7-5-2 Kusunoki-cho, Chuo-ku, Kobe 650-0017, Japan; nanaccoxox@gmail.com (N.F.); moriori@tg8.so-net.ne.jp (M.E.); smizob@med.kobe-u.ac.jp (S.M.); 6 Department of Anesthesiology, Toho University Ohashi Medical Center, 2-22-36 Ohashi, Meguro-ku, Tokyo 153-8515, Japan; maki@med.toho-u.ac.jp (Y.M.); ykotake@med.toho-u.ac.jp (Y.K.)

**Keywords:** extubation, ventilator liberation, mechanical ventilation, noninvasive respiratory support, prediction model, intensive care

## Abstract

Liberation from mechanical ventilation is of great importance owing to related complications from extended ventilation time. In this prospective multicenter study, we aimed to construct a versatile model for predicting extubation outcomes in critical care settings using obtainable physiological predictors. The study included patients who had been extubated after a successful 30 min spontaneous breathing trial (SBT). A multivariable logistic regression model was constructed to predict extubation outcomes (successful extubation without reintubation and uneventful extubation without reintubation or noninvasive respiratory support) using eight parameters: age, heart failure, respiratory disease, rapid shallow breathing index (RSBI), PaO_2_/FIO_2_, Glasgow Coma Scale score, fluid balance, and endotracheal suctioning episodes. Of 499 patients, 453 (90.8%) and 328 (65.7%) achieved successful and uneventful extubation, respectively. The areas under the curve for successful and uneventful extubation in the novel prediction model were 0.69 (95% confidence interval (CI), 0.62–0.77) and 0.70 (95% CI, 0.65–0.74), respectively, which were significantly higher than those in the conventional model solely using RSBI (0.58 (95% CI, 0.50–0.66) and 0.54 (95% CI, 0.49–0.60), *p* = 0.004 and <0.001, respectively). The model was validated using a bootstrap method, and an online application was developed for automatic calculation. Our model, which is based on a combination of generally obtainable parameters, established an accessible method for predicting extubation outcomes after a successful SBT.

## 1. Introduction

Extubation, which refers to the liberation of an intubated patient from invasive mechanical ventilation, is a critical stage during intensive care. The decision to extubate a patient is usually made after performing a weaning readiness test, wherein the patient undergoes spontaneous breathing with low levels of ventilatory assistance or through a T-piece [1]. Recent international guidelines recommend conducting a spontaneous breathing trial (SBT) before extubation [2,3]. However, extubation failure occurs in 6–19% of patients who successfully pass the SBT; therefore, making decisions regarding extubation remains a challenge [4,5].

Predictors of extubation outcomes have been comprehensively investigated, given the reported association between extubation failure and patient mortality [6]. Clinically significant predictors of extubation outcome include age, cardiopulmonary disorders (underlying cardiopulmonary disease and the occurrence of heart failure or pneumonia), rapid shallow breathing index ((RSBI), referring to the ratio of breathing frequency to tidal volume (f/VT)), PaO_2_/FIO_2_, and mental status evaluated using the Glasgow Coma Scale (GCS) score [5,6,7,8,9,10,11]. Moreover, recent cohort studies have demonstrated a positive effect of fluid balance [9,12] and a negative effect of excessive tracheobronchial secretion [11,13] on extubation outcomes. However, the abilities of these indicators to predict extubation outcomes have varied among studies, and evidence regarding their independent effects remains insufficient [14,15]. Based on the unified weaning readiness techniques, we hypothesized that a combination of previously reported, clinically important physiological predictors would allow us to construct the model for predicting extubation outcomes. Some groups have developed models that integrate multiple factors to predict extubation outcomes [4,16,17]. However, while practically available, these models were developed based on single-center observational studies and contained predictors that are considered subjective or that are not invariably measured in daily clinical practice. Thus, a generalized prediction model based on routinely obtained variables that are both objective and observable should be developed based on data from multiple centers.

We conducted a prospective multicenter observational study among critically ill patients who passed a 30 min SBT using a low fixed level of pressure support with positive end-expiratory pressure (PEEP). The study aimed to establish a versatile prediction model for extubation outcomes that utilizes prespecified clinically and bibliographically relevant and obtainable physiological predictors (designated the Prediction Of Successful Extubation (POSE) model). Currently, noninvasive respiratory support (noninvasive ventilation (NIV) or high-flow nasal oxygen (HFNO)) for sustained respiratory failure is widely performed after extubation [2,3,18,19]. Noninvasive respiratory support modalities effectively reduce the rate of reintubation [20,21], and predicting the potential need for such support is imperative to make the most effective use of limited medical resources. Therefore, we addressed the use of noninvasive respiratory support (without reintubation) and reintubation as extubation outcomes.

## 2. Materials and Methods

Ethics approval was obtained from the institutional ethics committees at each participating study site. The primary ethics committee was the Research Ethics Committee of Osaka University (approval number: 16526). The need for written informed consent was waived due to the observational design of this study, which consisted of routine care in the studied intensive care units (ICUs) based on weaning strategies recommended by nationwide consensus. This study was registered at ClinicalTrials.gov on 28 April 2017.

### 2.1. Study Population and Eligibility Criteria

Consecutive adult patients who had undergone invasive mechanical ventilation for more than 24 h and had been extubated following a successful SBT and cuff leak test (CLT) between 1 May 2017 and 30 April 2019 were enrolled in a prospective multicenter cohort. The cohort spanned multidisciplinary ICUs across five tertiary care hospitals in Japan. Each multidisciplinary ICU included 8 to 29 beds. Dedicated intensivists with backgrounds in anesthesiology, internal medicine, and emergency medicine managed mechanical ventilation strategies and made decisions regarding both weaning and extubation procedures. We excluded patients younger than 18 years, tracheotomized patients, patients who had died under mechanical ventilation or following withdrawal of support, those who discharged with mechanical ventilation, those who had been extubated without successful SBT or CLT as designated, and those who had undergone unplanned extubation or extracorporeal membrane oxygenation. Some patients in this study were included in a single-center study conducted in Osaka University [22].

### 2.2. Weaning and Extubation Procedures

All patients were screened every morning by intensivists and bedside nurses at a medical meeting, wherein patient-related issues were discussed and weaning decisions were made. Patients were extubated if they tolerated the designated SBT for 30 min with a PEEP of 5 cmH_2_O and pressure support of 5 cmH_2_O. Prior to extubation, a low risk of upper airway obstruction was confirmed based on negative CLT results, a cuff leak volume > 110 mL, and a percent cuff leak of >10% [23,24]. Details regarding the weaning readiness tests are provided in Table S1. After extubation, hemodynamic and respiratory parameters were continuously monitored for 48 h. Intensivists solely made decisions pertaining to patient reintubation and the use of noninvasive respiratory support without involving the research team based on standard practices. Patients were reintubated at the clinician’s discretion without a specific protocol if they exhibited cardiac arrest, refractory hypoxemia, severe hemodynamic instability without response to fluids and vasoactive drugs, persistent inability to remove excessive secretions, upper airway obstruction, agitation, or loss of consciousness.

### 2.3. Study Endpoints: The Outcomes That Were Predicted by the Prediction Model

The study endpoints were extubation outcomes following successful and unsuccessful extubation. Successful extubation was defined as the non-requirement of reintubation 48 h following extubation. Uneventful extubation was defined as the non-requirement of reintubation or noninvasive respiratory support 48 h following extubation.

### 2.4. Parameters for Developing the Prediction Model and Data Collection

We considered the objectivity and veracity of each parameter and discarded those that were not obtainable in common clinical practice by reviewing potential predictors of extubation outcomes cited in previous reports. The following eight predictors were selected as variables for the POSE model prior to patient enrollment: (1) age [5,6,7,8], (2) underlying or newly occurring heart failure [6,7,8], (3) underlying respiratory disease or pneumonia occurrence [7,9], (4) RSBI [9,10], (5) fluid balance during the previous 24 h [9], (6) PaO_2_/FIO_2_ [10], (7) GCS score [10,11], and (8) the number of endotracheal suctioning episodes during the previous 24 h as an indicator of the amount of tracheobronchial secretion [11,13,25]. We collected data related to patient characteristics including age, sex, body mass index, Acute Physiology and Chronic Health Evaluation II score, comorbidities, type of ICU admission, reason for intubation, and duration of mechanical ventilation. Underlying and new occurrence of heart failure was defined as a New York Heart Association functional classification of IV or left ventricular ejection fraction ≤ 40%, obtained from the comorbidity diagnosis and reason for mechanical ventilation. Underlying respiratory diseases included chronic obstructive pulmonary disease (COPD), asthma, and other respiratory diseases (restrictive or obstructive lung diseases) as comorbidities and reasons for mechanical ventilation. The occurrence of pneumonia was identified as both the primary reason for mechanical ventilation and pneumonia occurrence based on observations made during mechanical ventilation. Simultaneously, data related to the process of weaning toward extubation, physiological and laboratory parameters, and the process of care during the 24 h period prior to extubation (including fluid balance and the number of endotracheal suctioning episodes) were recorded. Arterial blood gas values and ventilation data were obtained at least 15 min after the commencement of a successful SBT to calculate the RSBI and PaO_2_/FIO_2_. Furthermore, the GCS score was assessed by the bedside nurse or intensivist prior to extubation, and a score of 1 point for the verbal component was recorded for intubated patients. Moreover, data regarding reintubation, the use of noninvasive respiratory support within 48 h after extubation, length of ICU stay and overall hospitalization, and mortality rates (ICU, 28-day, and hospital mortality) were recorded.

### 2.5. Statistical Analysis

Based on existing data for extubation outcomes and the event per variable formula [26], we originally determined that enrollment of 480 patients would be required to establish the prediction model using the eight factors mentioned above. Therefore, we designed an observational study across five ICUs that treated approximately 200–300 eligible patients per year.

To summarize baseline characteristics and process-of-care parameters, we calculated medians and interquartile ranges for continuous variables and proportions and counts for categorical variables. The first extubation attempts after more than 24 h of mechanical ventilation were analyzed for patients who received multiple series of mechanical ventilation. To construct the POSE model, we utilized a multivariable proportional odds logistic regression model with an ordinal categorical variable, consisting of the following categories as a function of the eight predictors: uneventful extubation (=3), use of noninvasive respiratory support within 48 h (without reintubation, =2), and reintubation within 48 h (=1). We introduced the three-way and two-way interaction terms between RSBI and PaO_2_/FIO_2_, between fluid balance and the number of endotracheal suctioning episodes during the previous 24 h. To improve the predictive performance of the model, these terms were also introduced in analyses among age, underlying or newly occurring heart failure, and underlying respiratory disease or pneumonia occurrence. To analyze the nonlinear effects on the results of extubation, we applied restricted cubic splines with three knots to continuous variables. Since continuous variables exhibit a generally nonlinear trend, we considered the nonlinear trend for all continuous variables using restricted-cubic-spline terms with three knots, which is expected to improve the prediction accuracy. All missing values were imputed using a multiple imputation method based on the predictive mean matching approach, and the number of the imputations was restricted to five. To describe the distribution of patient characteristics and process-of-care parameters between the event categories, we utilized Kruskal–Wallis and chi-square tests for continuous variables and categorical variables, respectively.

We used the Shiny R application to calculate the predicted probability of successful and uneventful extubations (POSE Calculator, https://statacademy.shinyapps.io/POSEmodel/ (accessed on 15 March 2022)). The calculator outputs the predicted probability by applying the input values of independent variables into the proportional odds logistic regression model estimated among the POSE study cohort.

To evaluate the predictive performance of the model, we calculated the calibration slopes using the bootstrap resampling method, which describes the association between the predicted probability and the observed probabilities of successful and uneventful extubations. Moreover, to compare the predictive performances of the POSE model and the traditional prediction model that utilizes the most representative parameter only (i.e., the RSBI, designated the RSBI model), we estimated the predictive performance of the latter using a univariable proportional odds logistic regression model that included RSBI as a predictor. As for the POSE model, the nonlinear effects were considered for the RSBI model. Subsequently, we calculated the predicted probabilities of successful and uneventful extubations and compared the predictive performances of the POSE and RSBI models based on the area under the receiver operating characteristic curve (AUC) for each model using the Delong method. For internal validation, we calculated the bootstrap mean and 95% coverage of the AUC and resampled 1000 times. Furthermore, we assessed the ability of the POSE model to improve discrimination when compared with the RSBI model using Net Reclassification Improvement (NRI) and Integrated Discrimination Improvement (IDI). Moreover, we performed a decision curve analysis to compare the clinical benefit of the decision to extubate based on each model. All statistical analyses were performed at a two-sided significance level of 5% using R version 4.0.3, R Foundation for Statistical Computing, Vienna, Austria (https://www.r-project.org/foundation/ (accessed on 15 March 2022)).

## 3. Results

### 3.1. Study Population and Clinical Characteristics

**Figure 1:**
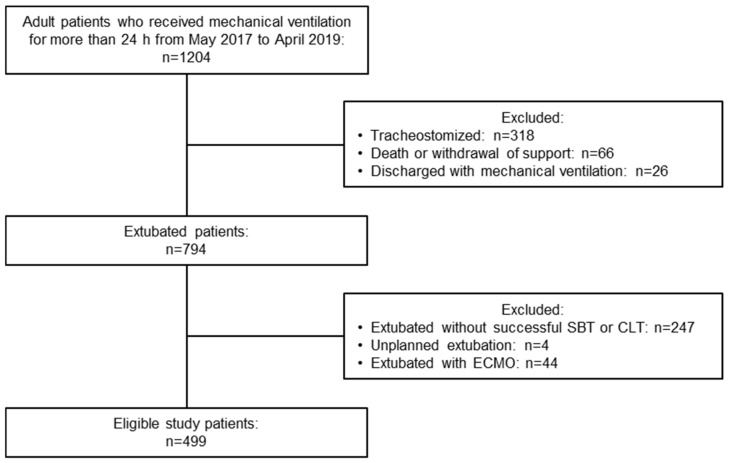
Flowchart of the study patients. SBT, spontaneous breathing trial; CLT, cuff leak test; ECMO, extracorporeal membrane oxygenation.

Over the 2-year study period, 1204 consecutive patients required invasive mechanical ventilation for more than 24 h. Of these, 499 patients fulfilled the study entry criteria and were extubated after tolerating the designated SBT for 30 min with a low risk of upper airway obstruction, as demonstrated in the CLT (Figure 1). Overall, 311 patients (62.3%) were males, with a median age of 69 years (interquartile range, 55–77 years) (Table 1). Moreover, 151 patients (30.3%) had underlying heart failure, and 112 (22.4%) had underlying respiratory failure (either COPD, asthma, or other respiratory diseases). Approximately 80% of all patients were intubated for respiratory failure, 10.0% were intubated for pneumonia, and 65.9% were in postoperative acute respiratory failure, defined as ineligible for weaning and extubation after surgery. Less than 5% of patients were intubated for neurological problems. The characteristics of the cohort study and parameters before extubation are provided in Table 1, Table 2 and Table S2.

Among the 499 patients with protocolized extubation, 46 patients (9.2%) were reintubated within 48 h. The other 453 patients (90.8%) achieved successful extubation without reintubation over the 48 h post extubation. Reintubation was performed at a median (interquartile range) of 10.0 (2.6–23.6) h after the extubation attempt (Table S3). The most common reason for reintubation was hypoxemia (63.0%), followed by excessive secretion (15.2%) and upper airway obstruction (10.9%).

The use of at least one noninvasive respiratory support measure was required in 149 patients (48 patients required NIV, 122 patients required HFNO, and 21 patients required both) within 48 h following extubation. Of these, 24 patients who were consequently reintubated were included in the classification of patients who required reintubation as an extubation outcome; thus, 125 patients were defined as those requiring noninvasive respiratory support (without reintubation). Accordingly, 328 patients (65.7%) achieved uneventful extubation without requiring reintubation or the use of noninvasive respiratory support.

### 3.2. Prediction Model and Online Calculator

The nonlinear relationships between the eight physiological factors and the estimated probability of a trend toward a favorable extubation outcome (from reintubation within 48 h to the use of noninvasive respiratory support within 48 h (without reintubation) to uneventful extubation) were described using restricted cubic splines in the ordinary multivariable logistic regression models adapted for potential interactions (Figure S1). As for the primary and secondary endpoints, the novel POSE model was established based on the multivariable analysis and depicted as an online calculator that provided the predictive incidence of extubation outcomes (successful and uneventful extubations) after a successful 30 min SBT (Figure S2).

### 3.3. Predictive Performance of the POSE Model

**Figure 2:**
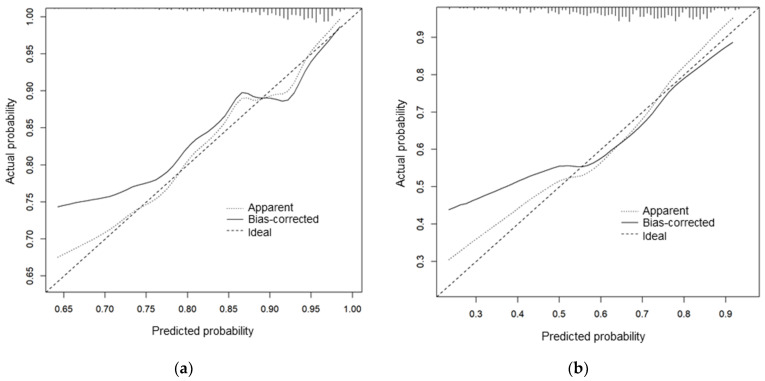
Calibration slopes for successful extubation (**a**) and uneventful extubation (**b**) based on the POSE model. The calibration slopes show the association between the actual probabilities of the events in the cohort (vertical axis) and the probabilities predicted by the POSE model (horizontal axis). We plotted the apparent line (Apparent) and the bias-corrected line (Bias-corrected). These lines indicated that the predicted probability does not deviate substantially from the actual probability of event occurrence. POSE, Prediction of Successful Extubation.

**Figure 3:**
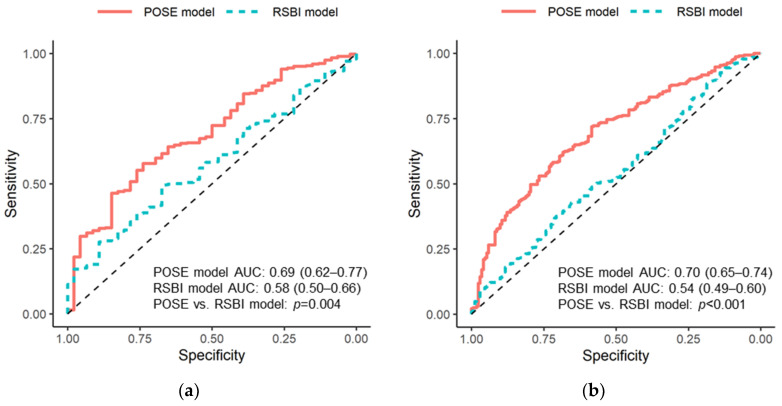
Receiver operating characteristic curves for the prediction models. The receiver operating characteristic curves for successful extubation (**a**) and uneventful extubation (**b**) for models based on eight predefined physiological factors (POSE model) and RSBI alone (RSBI model). The AUCs are shown for each model, and the *p*-values represent the intergroup differences. POSE, Prediction of Successful Extubation; AUC, area under the receiver operating characteristic curve; RSBI, rapid shallow breathing index.

**Figure 4:**
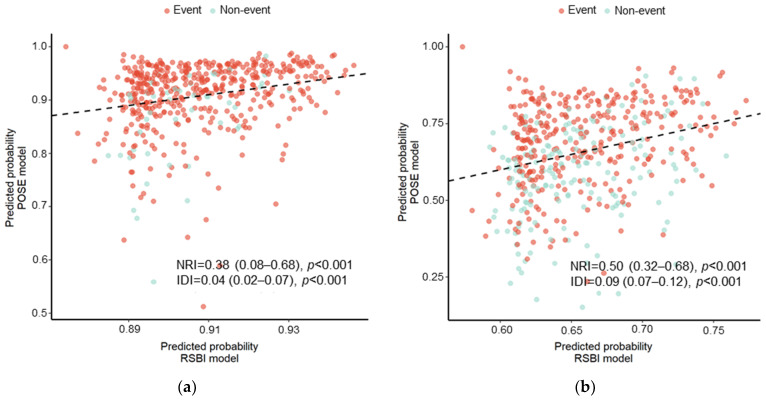
Predictive probabilities for successful extubation (**a**) and uneventful extubation (**b**). The inter-model differences between the novel prediction model based on eight predefined physiological factors (POSE model) and the conventional prediction model comprising RSBI alone (RSBI model) are demonstrated using NRI and IDI. Event indicates successful extubation (**a**) and uneventful extubation (**b**). The dashed line indicates the coincidence between the predicted probabilities of the POSE model (vertical axis) and the RSBI model (horizontal axis). The majority of patients with events fell above the dashed line, demonstrating that the POSE model predicts a higher probability of event occurrence than the RSBI model. POSE, Prediction of Successful Extubation; RSBI, rapid shallow breathing index; NRI, Net Reclassification Improvement; IDI, Integrated Discrimination Improvement.

**Figure 5:**
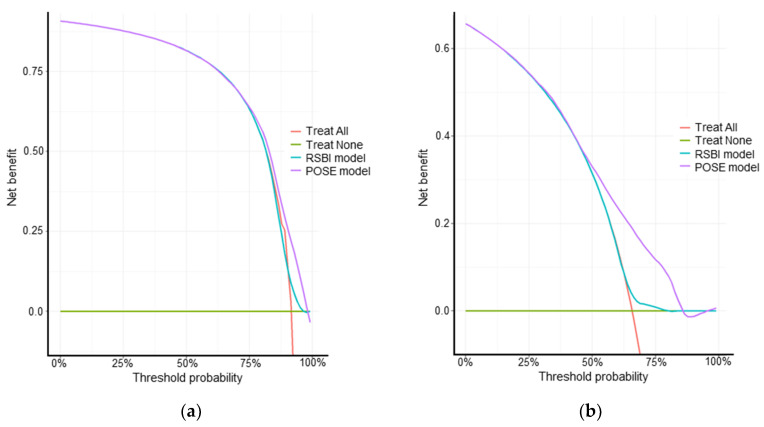
Decision curves for successful extubation (**a**) and uneventful extubation (**b**) predicted using each model. The decision curves show the net benefit for patients (vertical axis) when using each prediction model to make the decision regarding extubation at each threshold probability (horizontal axis). These results indicate that extubation decisions based on the POSE model may provide greater benefit than those based on the RSBI model, those based on the Treat-All approach (extubation for all patients), or those based on the Treat-None approach (no extubation). POSE, Prediction of Successful Extubation; RSBI, rapid shallow breathing index.

Calibration slopes for the POSE model indicated that the predicted probabilities did not deviate from the actual probabilities in the present cohort (Figure 2). Figure 3 shows the receiver operating characteristic curves predicting successful and uneventful extubations using the POSE and RSBI models in our study population. The AUCs for predicting successful extubation were 0.69 (95% confidence interval (CI), 0.62–0.77) in the POSE model and 0.58 (95% CI, 0.50–0.66) in the RSBI model. The AUCs for predicting uneventful extubation were 0.70 (95% CI, 0.65–0.74) in the POSE model and 0.54 (95% CI, 0.49–0.60) in the RSBI model. These two models demonstrated significant intergroup differences (*p* = 0.004 and *p* < 0.001 for the POSE and RSBI models, respectively). Moreover, the internal validation of the POSE model demonstrated excellent reproducibility. In bootstrapping analyses, the AUCs for successful and uneventful extubations were 0.69 (95% CI, 0.62–0.76) and 0.70 (95% CI, 0.65–0.74), respectively, in the POSE model. The predictive probabilities of the POSE and RSBI models are demonstrated in Figure 4. For the POSE, there were significant inter-model differences in NRI (0.38; 95% CI, 0.08–0.68; *p* < 0.001) and IDI (0.04; 95% CI, 0.02–0.07; *p* < 0.001). There were also significant inter-model differences in NRI (0.50; 95% CI, 0.32–0.68; *p* < 0.001) and IDI (0.09; 95% CI, 0.07–0.12; *p* < 0.001) for the prediction of uneventful extubation. The decision curves using the prediction models demonstrated that making the decision of the extubation based on the POSE model was better than those based on the RSBI model, especially when using higher thresholds (Figure 5).

## 4. Discussion

### 4.1. Key Findings

The present prospective multicenter cohort study relied on unified weaning readiness techniques to establish a usable prediction model for extubation outcomes, based on objective parameters that are readily available in general critical care settings. Compared with the classical prediction using the RSBI, the POSE model exhibited significantly improved predictive probability. Moreover, the online calculator facilitated the interactive prediction of extubation outcomes after a successful 30 min SBT.

### 4.2. Prediction of Extubation Outcome

Since the original report by Yang and Tobin in 1991 [27], the RSBI has been used as an instrumental factor in the prediction of extubation outcomes owing to the simplicity of the technique and avoidance of sophisticated equipment [14]. The usefulness of the RSBI has been widely acknowledged, and the breathing frequency that constitutes the RSBI has been generally included in the criteria for the SBT in current critical care settings. In a recent prospective single-center cohort study conducted in Brazil, the RSBI itself (threshold of RSBI < 105 breaths/min/L) was added to the criteria for extubation after a successful SBT [17], given the significant association between higher RSBI and an increased risk of extubation failure (odds ratio, 1.06; 95% CI, 1.02–1.10). Thus, RSBI is an indispensable factor for making extubation decisions in clinical practice; however, ventilatory support settings [28] and variation due to SBT initiation [29] can significantly influence the RSBI. Therefore, we utilized the RSBI value at least 15 min after the commencement of a successful SBT under a unified ventilatory support setting with other parameters to improve its predictive performance [9,30] when establishing the novel POSE model.

Developing a model for predicting extubation outcomes with applicable and easily obtainable objective predictors has been a challenge in clinical practice. In a retrospective, single-center observational study, Lai et al. developed a nomogram for predicting successful extubation [4]. Based on the clinical records of patients extubated after a successful SBT, multivariable logistic regression identified three significant respiratory factors, including RSBI. However, the other factors were considered subjective and related to airway patency. Another predictive model was introduced by Kuo et al. after a prospective observation of 121 patients [16]. The abovementioned study utilized respiratory monitoring during the unified SBT and developed a decision–support system that exhibited accurate predictive performance with predefined parameters. However, a sophisticated computer-based system that relies on confidential algorithms is indispensable. Most recently, in a single-center cohort study by Baptistella et al., a prediction model has been reported [17]. Similar to the present study, adult patients who passed a 30 min T-piece SBT and CLT were included in that study. In addition to patient characteristics, including comorbidities and general clinical signs and ventilator parameters such as RSBI, detailed respiratory information including dynamic lung compliance and assessment of muscle strength graded on the Medical Research Council scale were also collated. Significant parameters from the univariate logistic analysis in a derivation cohort of 110 patients were determined. These parameters, including RSBI in SBT, dynamic lung compliance, duration of mechanical ventilation, muscle strength, estimated GCS, hematocrit, serum creatinine, and presence of neurologic comorbidity, were used to develop the model with an AUC of 0.875. This model was validated in 83 subsequent patients. Although the model has not been evaluated for its external validity at other institutions, it might be practically used in patients whose detailed respiratory parameters and muscle strength assessments are available. In order to establish a versatile prediction model for critically ill patients, the present study was designed with objective parameters that can be obtained in common clinical practice, which were used to develop an interactive online calculator. The eight physiological factors, including RSBI, were precedingly designated based on existing studies and clinical importance to avoid bias caused by exploratory analyses [31,32]. The ordinary multivariable logistic regression models in this cohort demonstrated that the association between each factor and extubation outcome was plausible as an overall trend.

### 4.3. Implications of Study Findings

Our study findings suggest that the combination of commonly available physiological factors significantly improved the ability to predict extubation outcomes after a successful 30 min SBT under unified ventilatory support settings. Our approach can incorporate the nonlinear interactions between predictors and provide convenient prediction online. Though the developed model requires further external validation in a larger population, clinicians and future studies can use this model to identify patients at a high risk of reintubation or those in need of noninvasive respiratory support.

### 4.4. Strengths and Limitations

The strength of this multicenter prospective study was that it established an accessible prediction model for complex extubation outcomes, including the need for noninvasive respiratory support, using objective and easily available physiological parameters based on the rigorous SBT assessment used in general ICU settings. However, our study had some limitations. First, although this was a multicenter study conducted among patients in general ICUs, more than half of the eligible patients were postoperative. This may represent a potential source of heterogeneity. Second, although the SBT and CLT assessments were protocolized, the attending clinicians made the final decision to extubate or reintubate patients. The standard criteria for administering noninvasive respiratory support in Japan are based on an observed respiratory status of progressive deterioration using mask oxygenation, with PaCO_2_ > 45 mmHg, pH < 7.35, and PaO_2_/FIO_2_ < 200 mmHg for NIV and on an SpO_2_ < 93% for HFNO, which clinicians at each institution use to form comprehensive judgments. This might have caused selection bias, and unmeasured confounders might have influenced extubation outcomes. Meanwhile, this study recorded observations for 48 h after extubation when assessing extubation outcomes. Subsequent processes were not included, and the impact of the medium-term period on patient outcomes was not assessed. However, the RSBI at extubation [17,33,34], the use of noninvasive respiratory support after extubation [35,36], and the reintubation rates [37,38] were comparable with those described in other recent studies, suggesting that our cohort was representative of current clinical practice. These findings suggest that our model can enable adaptable and robust extubation outcome prediction. Third, in the evaluation of GCS, the verbal component may be predicted (e.g., the estimated GCS) in clinical practice [39,40]. However, in this study, we used a consistent value for the verbal component of GCS to reduce potential observer bias. Fourth, since the predictive model contains substantial nonlinear and nonadditive terms, the model equations, including intercepts and coefficients for covariates, cannot be simply formulated in mathematical terms. Therefore, restricted cubic splines were included to provide an intuitive explanation regarding the impact of each covariate on the outcome variable. Fifth, our analysis did not include an external dataset for validation of the prediction model. The predictive performance was assessed via internal validation based on the bootstrap resampling approach. Furthermore, although the novel prediction model involved different predictors than previously reported models, we did not compare predictive performance among these models.

## 5. Conclusions

The proposed POSE model, developed based on eight predefined physiological variables, offers a relevant and versatile tool for predicting extubation outcomes among critically ill patients with a successful SBT. Further prospective studies with larger sample sizes are warranted to confirm the external validity and evaluate the performance of this model.

## Figures and Tables

**Table 1:** Intubation characteristics of patients in the cohort study stratified by extubation outcomes.

Characteristics	Total Cohort (*n* = 499)	Uneventful Extubation (*n* = 328) ^a^	Noninvasive Respiratory Support (*n* = 125) ^b^	Reintubation (*n* = 46)	*p* Value	Missing (%)
Age, years	69 (55–77)	69 (55–77)	69 (54–78)	70 (63–76)	0.913	0
Male sex, *n* (%)	311 (62.3%)	201 (61.3%)	79 (63.2%)	31 (67.4%)	0.706	0
Body mass index, kg/m^2^	22.5 (19.8–25.4)	22.2 (19.8–25.2)	23.7 (20.1–26.7)	21.0 (19.0–24.0)	0.025	0
APACHE II score	19 (14–24)	19 (14–23)	19 (14–25)	18 (14–22)	0.386	0
Comorbidity, *n* (%)						
Heart failure	151 (30.3%)	94 (28.7%)	45 (36.0%)	12 (26.1%)	0.194	0
COPD	33 (6.6%)	18 (5.5%)	11 (8.8%)	4 (8.7%)	0.375	0
Asthma	23 (4.6%)	15 (4.6%)	6 (4.8%)	2 (4.3%)	0.991	0
Other respiratory diseases	66 (13.2%)	37 (11.3%)	18 (14.4%)	11 (23.9%)	0.300	0
Diabetes mellitus	130 (26.1%)	82 (25.0%)	33 (26.4%)	15 (32.6%)	0.543	0
Chronic kidney disease	101 (20.2%)	66 (20.1%)	23 (18.4%)	12 (26.1%)	0.538	0
Surgical ICU admission, *n* (%)	355 (71.1%)	224 (68.3%)	93 (74.4%)	38 (82.6%)	0.087	0
Reason for mechanical ventilation, *n* (%)						
Asthma	2 (0.4%)	2 (0.6%)	0 (0.0%)	0 (0.0%)	0.096	0
COPD	0 (0.0%)	0 (0.0%)	0 (0.0%)	0 (0.0%)		
Pneumonia	50 (10.0%)	33 (10.0%)	9 (7.2%)	8 (17.4%)		
ARDS	4 (0.8%)	3 (0.9%)	1 (0.8%)	0 (0.0%)		
Postoperative acute respiratory failure	329 (65.9%)	207 (63.1%)	88 (70.4%)	34 (73.9%)		
Upper airway obstruction	4 (0.8%)	3 (0.9%)	1 (0.8%)	0 (0.0%)		
Other causes of respiratory failure	17 (3.4%)	10 (3.0%)	6 (4.8%)	1 (2.2%)		
Sepsis	23 (4.6%)	17 (5.2%)	4 (3.2%)	2 (4.3%)		
Heart failure	29 (5.8%)	21 (6.4%)	8 (6.4%)	0 (0.0%)		
Coma	12 (2.4%)	8 (2.4%)	3 (2.4%)	1 (2.2%)		
Neurological disease	10 (2.0%)	9 (2.7%)	1 (0.8%)	0 (0.0%)		
Trauma	1 (0.2%)	0 (0.0%)	1 (0.8%)	0 (0.0%)		
Cardiac arrest	18 (3.6%)	15 (4.6%)	3 (2.4%)	0 (0.0%)		

Data are expressed as medians (interquartile range) or *n* (%). ^a^ Uneventful extubation was defined as the non-requirement of reintubation or noninvasive respiratory support within 48 h post extubation. ^b^ Patients in the noninvasive respiratory support group were administered NIV or HFNO within 48 h post extubation (without reintubation). APACHE Acute Physiology and Chronic Health Evaluation, COPD chronic obstructive pulmonary disease, ICU intensive care unit, ARDS acute respiratory distress syndrome, NIV noninvasive ventilation, HFNO high-flow nasal oxygen.

**Table 2:** Data prior to extubation and patient outcomes.

Variables	Total Cohort	Uneventful Extubation ^a^	Noninvasive Respiratory Support ^b^	Reintubation	*p* Value	Missing (%)
Duration of mechanical ventilation, h	83.5 (45.8–139.2)	68.9 (44.1–119.9)	92.4 (49.3–184.8)	99.7 (66.2–164.6)	<0.001	0
Cardiopulmonary disorders prior to extubation, *n* (%)						
Underlying or new occurrence of heart failure ^c^	159 (31.9%)	100 (30.5%)	47 (37.6%)	12 (26.1%)	0.236	0
Underlying respiratory disease or occurrence of pneumonia ^c^	183 (36.7%)	111 (33.8%)	48 (38.4%)	24 (52.2%)	0.049	0
ABG levels and respiratory data during successful SBT						
pH	7.43 (7.40–7.46)	7.43 (7.40–7.46)	7.44 (7.41–7.48)	7.45 (7.41–7.46)	0.165	0
PaCO_2_, mmHg	40.5 (36.8–44.2)	40.5 (36.9–43.8)	40.0 (36.1–44.0)	43.3 (38.4–46.1)	0.050	0
PaO_2_/FIO_2_, mmHg	300 (242–367)	311 (259–381)	260 (220–340)	314 (227–354)	<0.001	0
SpO_2_, %	98 (97–100)	99 (98–100)	98 (97–99)	98 (96–99)	0.007	0
RSBI, breaths/min/L	41.8 (31.3–55.7)	40.3 (30.7–55.0)	41.8 (31.1–56.9)	45.1 (36.5–56.3)	0.141	0
Parameters at extubation						
SOFA score	8 (6–10)	8 (6–10)	9 (7–11)	8 (5–10)	0.002	0
Fluid balance during the previous 24 h, mL	−296 (−1053–400)	−313 (−1032–498)	−247 (−1059–343)	−412 (−1198–276)	0.659	0
GCS score, point	11 (10–11)	11 (10–11)	11 (10–11)	10 (10–11)	0.314	0
Number of endotracheal suctioning episodes during the previous 24 h	12 (9–16)	12 (9–14)	13 (9–16)	15 (11–18)	0.659	0
Patient outcomes						
ICU length of stay, d	8 (5–14)	7 (4–11)	10 (6–17)	17 (13–27)	<0.001	0
Hospital length of stay, d	46 (28–83)	40 (27–77)	48 (29–87)	75 (49–127)	<0.001	0
28-day mortality, *n* (%)	9 (1.8%)	5 (1.5%)	3 (2.4%)	1 (2.2%)	0.806	0
ICU mortality, *n* (%)	13 (2.6%)	5 (1.5%)	7 (5.6%)	1 (2.2%)	0.051	0
Hospital mortality, *n* (%)	43 (8.6%)	23 (7.0%)	14 (11.2%)	6 (13.0%)	0.194	0

Data are expressed as medians (interquartile range) or *n* (%). ^a^ Uneventful extubation was defined as the non-requirement of reintubation or noninvasive respiratory support within 48 h post extubation. ^b^ Patients in the noninvasive respiratory support group were administered NIV or HFNO within 48 h post extubation (without reintubation). ^c^ Underlying and new occurrence of heart failure was assessed as a comorbidity and as a reason for mechanical ventilation. Underlying respiratory diseases included comorbidities, and the reasons for mechanical ventilation and the occurrence of pneumonia were based on observations made during mechanical ventilation. ABG arterial blood gas, SBT spontaneous breathing trial, RSBI rapid shallow breathing index, SOFA Sequential Organ Failure Assessment, GCS Glasgow Coma Scale, ICU intensive care unit.

## Data Availability

The datasets used and/or analyzed during the current study are available from the corresponding author on reasonable request.

## Supplementary Materials

The following supporting information can be downloaded at: https://www.mdpi.com/article/10.3390/jcm11092520/s1. Table S1. Details of weaning readiness tests; Table S2. Process of weaning and additional data prior to extubation; Table S3. Respiratory support within 48 h of extubation; Figure S1. Effects of the eight predefined physiological factors on extubation outcomes within 48 h; Figure S2. A screenshot of the online calculator that provides the predicted probabilities of successful and uneventful extubations after a successful 30 min SBT for patients with a low risk of upper airway obstruction.
