# Supplementary material for: Prediction Model of Extubation Outcomes in Critically Ill Patients: A Multicenter Prospective Cohort Study
Source: J Clin Med. 2022 Apr 29;11(9):2520. doi: 10.3390/jcm11092520 (PMC9102390; doi:10.3390/jcm11092520)
Supplement: Supplementary file 1 [file jcm-11-02520-s001.zip › jcm-1676415-supplementary.pdf]

Supplementary Materials

Table S1. Details of weaning readiness tests

Table S2. Process of weaning and additional data prior to extubation

Table S3. Respiratory support within 48 hours of extubation

Figure S1. Effects of the eight predefined physiological factors on extubation outcomes within 48 h

Figure S2. A screenshot of the online calculator that provides the predicted probabilities of successful and uneventful extubations after a successful 30-min SBT for patients with a low risk of upper airway obstruction.

Table S1. Details of weaning readiness tests

**Spontaneous breathing trial (SBT)**<br>Patients were determined to qualify for SBT when they exhibited improvement in or a resolution of the underlying respiratory failure or cause of mechanical ventilation, sufficient oxygenation and inspiratory effort, hemodynamic stability, and an adequate respiratory pattern, in accordance with standard clinical practices for weaning strategies driven by national consensus. The SBT was abandoned if a patient met the following interruption criteria: respiratory rate ≥30 breaths/min, significant deterioration of oxygenation (SpO_2 <94%, PaO_2 <70 mmHg), deterioration of hemodynamic stability (heart rate ≥140 beats/min, newly developed arrhythmia, or signs of myocardial ischemia), a significant increase in blood pressure after SBT initiation, or appearance of respiratory distress (defined as paradoxical breathing, use of accessory muscles, sweating, or agitation). Suitability for extubation was determined based on the results of a 30-min SBT with a positive end-expiratory pressure of 5 cmH_2O and pressure support of 5 cmH_2O.
**Cuff leak test (CLT)**<br>Prior to endotracheal extubation, the patient underwent intraoral and intratracheal suctioning in preparation for the CLT. Assist-control ventilation mode was used for the CLT, in accordance with the standard clinical practices of the participating institutions, and the displayed expiratory tidal volume was monitored while the endotracheal tube cuff was in the inflated position. The cuff was then deflated, and the following six respiratory cycles were monitored to eliminate erroneous values. The difference between the expiratory tidal volume with the cuff inflated and the average of the three lowest expiratory tidal volumes during the six respiratory cycles with the cuff deflated was defined as the cuff leak volume. Additionally, the percent cuff leak, which was defined as the percentage of the cuff leak volume relative to the expiratory tidal volume with the cuff inflated, was calculated. A low risk of upper airway obstruction was confirmed based on a negative CLT result, a cuff leak volume >110 mL, and a percent of cuff leak >10%.

SBT: spontaneous breathing trial, *CLT:* cuff leak test

Table S2. Process of weaning and additional data prior to extubation

Variables	Total cohort (n=499)	Uneventful extubation (n=328) [a]	Noninvasive respiratory support (n=125) [b]	Reintubation (n=46)	P value	Missing (%)
Process of weaning						
Method of weaning, n (%)						
Once-daily SBT	28 (5.6%)	18 (5.5%)	9 (7.2%)	1 (2.2%)	0.398	0
Multiple daily SBTs	3 (0.6%)	1 (0.3%)	1 (0.8%)	1 (2.2%)		
Gradual reduction of pressure support	468 (93.8%)	309 (94.2%)	115 (92.0%)	44 (95.7%)		
SBT attempts	1 (1–1)	1 (1–1)	1 (1–1)	1 (1–1)	0.422	0
Data during 24 h prior to the extubation						
Fever (≥38°C), n (%)	167 (33.5%)	108 (32.9%)	45 (36.0%)	14 (30.4%)	0.743	0
Use of sedatives, n (%)	466 (93.4%)	304 (92.7%)	119 (95.2%)	43 (93.5%)	0.628	0
Use of neuromuscular blocker, n (%)	1 (0.2%)	1 (0.3%)	0 (0.0%)	0 (0.0%)	0.77	0
Use of opioids, n (%)	342 (68.5%)	222 (67.7%)	89 (71.2%)	31 (67.4%)	0.76	0
Use of steroids (methylprednisolone ≥20 mg), n (%)	72 (14.4%)	47 (14.3%)	18 (14.4%)	7 (15.2%)	0.987	0
Use of vasopressors, n (%)	301 (60.3%)	197 (60.1%)	76 (60.8%)	28 (60.9%)	0.989	0
CVP, mmHg	8 (6–10)	8 (6–10)	9 (6–11)	8 (7–11)	0.327	15
Laboratory data						
White blood cells, /µL	9,600 (7,165–12,320)	9,645 (7,413–12,563)	9,720 (7,100–12,300)	8,750 (6,855–10,330)	0.105	0
Hemoglobin, g/dL	10.4 (9.4–11.4)	10.4 (9.5–11.4)	10.4 (9.3–11.4)	10.2 (9.3–11.2)	0.724	0
Platelets, mm^3	106,000 (75,000–166,000)	106,000 (75,500–165,000)	97,500 (69,000–170,250)	103,000 (79,750–171,000)	0.643	0.4
Creatinine, µmol/L	84.9 (58.3–154.3)	82.2 (58.3–155.8)	90.2 (61.9–159.1)	80.9 (53.9–119.1)	0.428	0
Lactate, mmol/L	1.0 (0.8–1.3)	1.0 (0.8–1.3)	1.0 (0.8–1.3)	0.9 (0.7–1.1)	0.158	0
Time from SBT to extubation, h	1.1 (0.5–2.9)	1.1 (0.5–2.8)	1.2 (0.5–3.7)	1.2 (0.6–2.5)	0.832	0

Data are expressed as medians (interquartile range) or n (%).

[a] Uneventful extubation was defined as the non-requirement of reintubation or noninvasive respiratory support within 48 h post extubation.

[b] Patients in the noninvasive respiratory support group were administered NIV or HFNO within 48 h post extubation (without reintubation).

SBT, spontaneous breathing trial, *CVP*, central venous pressure

Table S3. Respiratory support within 48 hours of extubation

Variables	Total cohort	Uneventful extubation [a]	Noninvasive respiratory support [b]	Reintubation	Missing (%)
NIV use, n (%)	48 (9.6%)	0 (0%)	37 (29.6%)	11 (23.9%)	0
Cause of NIV use, n (%)					
Refractory hypoxemia	18 (3.6%)	0 (0%)	12 (9.6%)	7 (15.2%)	
Increased breathing effort	10 (2.0%)	0 (0%)	7 (5.6%)	3 (6.5%)	
Heart failure	4 (0.8%)	0 (0%)	3 (2.4%)	1 (2.2%)	
Prophylactic	15 (3.0%)	0 (0%)	15 (12.0%)	0 (0%)	
HFNO use, n (%)	122 (24.4%)	0 (0%)	103 (82.4%)	19 (41.3%)	0
Cause of HFNO use, n (%)					
Refractory hypoxemia	30 (6.0%)	0 (0%)	21 (16.8%)	9 (19.6%)	
Increase of work of breathing	14 (2.8%)	0 (0%)	9 (7.2%)	5 (10.9%)	
Heart failure	6 (1.2%)	0 (0%)	6 (4.8%)	0 (0%)	
Prophylactic	72 (14.4%)	0 (0%)	67 (53.6%)	5 (10.9%)	
Use of NIV or HFNO, n (%)	149 (29.9%)	0 (0%)	125 (100%)	24 (52.2%)	0
Time from extubation to reintubation, h	10.0 (2.6–23.6)	–	–	10.0 (2.6–23.6)	
Cause of reintubation, n (%)					
Cardiac arrest	1 (0.2%)	0 (0%)	0 (0%)	1 (2.2%)	
Refractory hypoxemia	29 (5.8%)	0 (0%)	0 (0%)	29 (63.0%)	
Hemodynamic instability	2 (0.4%)	0 (0%)	0 (0%)	2 (4.3%)	
Excessive secretion	7 (1.4%)	0 (0%)	0 (0%)	7 (15.2%)	
Upper airway obstruction	5 (1.0%)	0 (0%)	0 (0%)	5 (10.9%)	
Agitation	1 (0.2%)	0 (0%)	0 (0%)	1 (2.2%)	
Loss of consciousness	1 (0.2%)	0 (0%)	0 (0%)	1 (2.2%)	

Data are expressed as medians (interquartile range) or n (%).
[a] Uneventful extubation was defined as the non-requirement of reintubation or noninvasive respiratory support within 48 h post extubation.
[b] Patients in the noninvasive respiratory support group were administered NIV or HFNO within 48 h post extubation (without reintubation).
NIV: noninvasive ventilation, *HFNO*: high-flow nasal oxygen

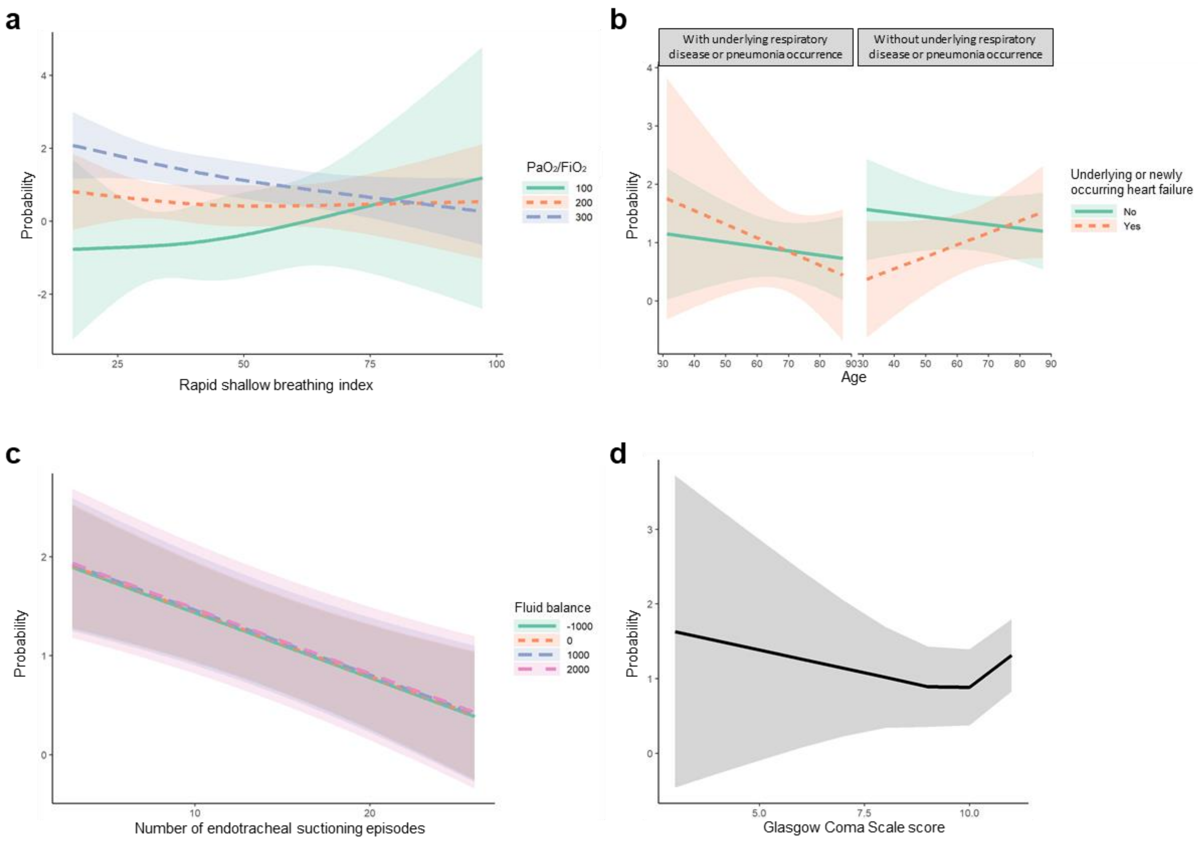

Figure S1. Effects of the eight predefined physiological factors on extubation outcomes within 48 h. Probability of a trend towards a favorable extubation outcome (from reintubation to the use of noninvasive respiratory support [without reintubation] to uneventful extubation) according to the eight designated physiological predictors: rapid shallow breathing index and PaO_2/FIO_2 (**a**); age, underlying or newly occurring heart failure, and underlying respiratory disease or pneumonia occurrence (**b**); fluid balance and the number of endotracheal suctioning episodes during the previous 24 h (**c**); and Glasgow Coma Scale score (**d**). Four hundred fifty-six patients (91.4%) had $PaO_2/FIO_2 \geq 200$, and 486 patients (97.4%) had Glasgow Coma Scale scores ≥8 points.

Figure S2. A screenshot of the online calculator that provides the predicted probabilities of successful and uneventful extubations after a successful 30-min SBT for patients with a low risk of upper airway obstruction.

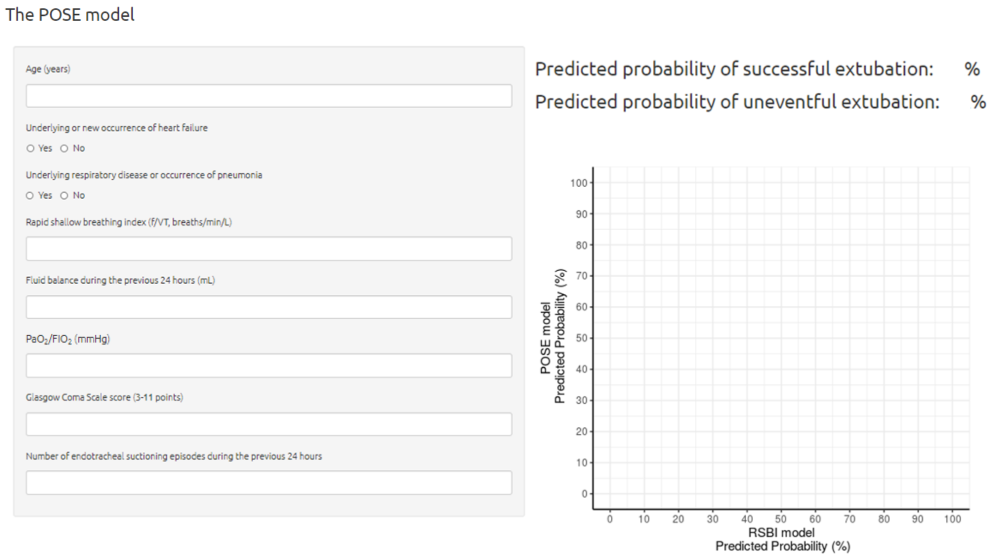

The online calculator (POSE model), resulting from the ordinary multivariable logistic regression model for the cohort study, automatically estimates the predictive probabilities of successful and uneventful extubations when the variables and choices of the eight factors for adult patients on mechanical ventilation with low risk of upper airway obstruction, who had passed the SBT for 30 min with pressure support ventilation (PEEP, 5 cmH_2O; pressure support, 5 cmH_2O), are entered. Successful extubation was defined as the non-requirement of reintubation within 48 h post extubation, and uneventful extubation was defined as the non-requirement of reintubation or noninvasive respiratory support 48 h post extubation. For reference, the prediction using the traditional prediction model comprising a single RSBI (RSBI model) is also shown in the graph.

SBT, spontaneous breathing trial; POSE, Prediction of Successful Extubation; RSBI, rapid shallow breathing index; PEEP, positive end-expiratory pressure.
